# Cardiovascular disease in adults with a history of out-of-home care during childhood: a systematic review and meta-analysis of prospective cohort studies

**DOI:** 10.1016/j.lanepe.2024.100984

**Published:** 2024-07-09

**Authors:** G. David Batty, Mika Kivimäki, Ylva B. Almquist, Johan G. Eriksson, Mika Gissler, Emmanuel S. Gnanamanickam, Mark Hamer, Josephine Jackisch, Hee-Soon Juon, Markus Keski-Säntti, Chaiquan Li, Tuija M. Mikkola, Emily Murray, Amanda Sacker, Leonie Segal, Philipp Frank

**Affiliations:** a Department of Epidemiology and Public Health, University College London, London, UK; b Brain Sciences, University College London, London, UK; c Clinicum, Faculty of Medicine, University of Helsinki, Helsinki, Finland; d Centre for Health Equity Studies, Stockholm University, Stockholm, Sweden; e Singapore Institute for Clinical Sciences, Singapore; f Department of Obstetrics & Gynaecology and Human Potential Translational Research Programme, National University of Singapore, Singapore; g Folkhälsan Research Center, Helsinki, Finland; h Department of General Practice and Primary Health Care, University of Helsinki, Helsinki, Finland; i Department of Knowledge Brokers, Finnish Institute for Health and Welfare, Helsinki, Finland; j Academic Primary Health Care Centre, Region Stockholm, Sweden; k Department of Molecular Medicine and Surgery, Karolinska Institutet, Stockholm, Sweden; l Health Economics and Social Policy Group, Allied Health & Human Performance, University of South Australia, Adelaide, Australia; m Division of Surgery Interventional Science, University College London, London, UK; n Department of Medical Oncology, Thomas Jefferson University, Philadelphia, USA; o Department of Epidemiology and Biostatistics, Peking University Health Science Center, Peking, China; p Folkhälsan Research Center, Helsinki, Finland; q Population Health Unit, Finnish Institute for Health and Welfare, Helsinki, Finland; r Institute of Public Health and Wellbeing, University of Essex, Colchester, UK

**Keywords:** Cardiovascular disease, Cohort study, Meta-analysis, Out-of-home care, Systematic review

## Abstract

**Background:**

While individuals who were separated from their biological family and placed into the care of the state during childhood (out-of-home care) are more prone to developing selected adverse health problems in adulthood, their risk of cardiovascular disease is uncertain. Our aim was to explore this association by pooling published and unpublished results from prospective cohort studies.

**Methods:**

We used two approaches to identifying relevant data on childhood care and adult cardiovascular disease (PROSPERO registration CRD42021254665). First, to locate published studies, we searched PubMed (Medline) until November 2023. Second, with the objective of identifying unpublished studies with the potential to address the present research question, we scrutinised retrieved reviews on childhood out-of-home care and other adult health outcomes. Included studies were required to satisfy three criteria: a cohort study in which the assessment of care was made prospectively pre-adulthood (in the avoidance of recall bias); data on an unexposed comparator group were available (for the computation of relative risk); and a diagnosis of adult cardiovascular disease events (coronary heart disease, stroke, or their combination) had been made (as opposed to risk factors only). Collaborating investigators provided study-specific estimates which were aggregated using random-effects meta-analysis. The Newcastle-Ottawa Scale was used to assess individual study quality.

**Findings:**

Twelve studies (2 published, 10 unpublished) met the inclusion criteria, and investigators from nine provided viable results, including updated analyses of the published studies. Studies comprised 611,601 individuals (301,129 women) from the US, UK, Sweden, Finland, and Australia. Five of the nine studies were judged to be of higher methodological quality. Relative to the unexposed, individuals with a care placement during childhood had a 51% greater risk of cardiovascular disease in adulthood (summary rate ratio after age- and sex-adjustment [95% confidence interval]: 1.51 [1.22, 1.86]; range of study-specific estimates: 1.07 to 2.06; *I*^*2*^ = 69%, p = 0.001). This association was attenuated but persisted after adjustment for socioeconomic status in childhood (8 studies; 1.41 [1.15, 1.72]) and adulthood (9 studies, 1.29 [1.11, 1.51]).

**Interpretation:**

Our findings show that individuals with experience of out-of-home care in childhood have a moderately raised risk of cardiovascular disease in adulthood.

**Funding:**

10.13039/501100000265Medical Research Council; 10.13039/100000049National Institute on Aging; 10.13039/100010269Wellcome Trust.

**Research in context**

**Evidence before this study**

There is growing evidence that individuals who were separated from their biological family and placed into the care of the state during childhood (out-of-home care) are more prone to developing selected physical and mental ill-health events in adulthood, however, their risk of cardiovascular disease is uncertain. A search of electronic databases to November 2023 yielded no systematic review or meta-analysis. The only two relevant individual published studies reported discordant findings.

**Added value of this study**

By scrutinising retrieved reviews of the impact of childhood out-of-home care on an array of adult health outcomes, we identified studies with the potential to examine the association between childhood care and adult cardiovascular disease events. Investigators from 7 studies provided these previously unpublished results and, on aggregating them alongside updated analyses from the 2 published studies, we found that, relative to their unexposed peers, adults with experience of care earlier in life had a 50% greater risk of cardiovascular disease. There was evidence that this relationship was partially mediated by socioeconomic status in adulthood.

**Implications of all the available evidence**

This meta-analysis suggests that, alongside the array of well-documented unfavourable social, behavioural, and health outcomes in adulthood, children experiencing out-of-home care may additionally have a higher burden of later cardiovascular disease.

## Introduction

Although decades-long progress in cardiovascular disease epidemiology has led to the identification of a series of modifiable risk factors,^1^ their measurement in middle- and older-age populations does not fully explain the occurrence of the condition.^2^^,^^3^ This raises the possibility that cardiovascular disease may have its origins in early life. Cohort studies with extended event surveillance have shown that individuals who were overweight, smoked cigarettes, or had higher levels of blood pressure and blood cholesterol in childhood or adolescence were more likely to develop atherosclerotic phenotypes^4^, ^5^, ^6^ and be diagnosed with cardiovascular disease^7^, ^8^, ^9^, ^10^, ^11^, ^12^, ^13^, ^14^ in adulthood.

Whereas there is growing evidence implicating these pre-adult physiological and behavioural risk factors in the aetiology of adult cardiovascular disease, the role of early life psychosocial characteristics is less certain. An increasingly examined exposure in this context is early life adversity. Denoted by an array of characteristics, including maltreatment (e.g., abuse or neglect by family or other trusted adults), parental loss or the threat thereof (e.g., divorce, incarceration), and a stressful home environment (e.g., parental mental illness, addiction),^15^ there is a strong *prima facie* case implicating childhood adversity in the development of adult cardiovascular disease. That is, relative to unaffected population controls, people experiencing childhood adversity subsequently have a greater prevalence of cardiovascular disease risk factors, including lifestyle indices such as cigarette smoking, heavy alcohol intake, obesity, and illicit drug use,^16^ and are more likely to be socioeconomically disadvantaged, as evidenced by higher levels of unemployment, lower occupational prestige, and modest educational attainment.^17^

Removal from the biological family into the apparently safer milieu of out-of-home care—also referred to as state care, public care, being looked-after, social care, or substitute care—most commonly in response to significant harm or the risk thereof, represents one of the more severe components of childhood adversity.^15^ As such, of the adversity indicators, an association for out-of-home care with later cardiovascular disease events would be anticipated but has been little-tested. In a recent meta-analysis of prospective studies we have shown that adults with a history of out-of-home care in childhood experience a doubling in the risk of premature mortality.^18^ While this gradient for total mortality was partly ascribed to a tripling in the occurrence of suicide in adults exposed to early life care,^18^ it is plausible that common chronic diseases in adulthood, specifically cardiovascular disease, might also contribute. With the two existing studies on pre-adult care and cardiovascular disease reaching discordant findings,^19^^,^^20^ the status of this relationship is uncertain.

A methodological concern in studies examining the risk of a given health outcomes in people experiencing pre-adult adversity, including out-of-home care, is the mode of exposure measurement. As exemplified in the progenitor Adverse Childhood Experiences Study,^21^ and in systematic reviews of the evidence base,^22^ the large majority of studies capture early life adversity in middle- or older-aged participants.^15^ Based on an aggregation of 20 studies which explored the validity of childhood prospective assessment of maltreatment—the gold standard in this context—against such distant recall of the same in later adulthood, there was low agreement.^23^ This may have important ramifications. For instance, in one study prospective measurement of childhood overcrowding revealed no association with adult respiratory disease, whereas for the retrospectively-captured data, higher levels actually appeared to confer protection against the same outcome.^24^ Similarly discordant were results from a Finnish study in which vascular disease was the outcome of interest.^25^

The purpose of the present systematic review and meta-analysis therefore is to add to the evidence base on early life adversity and adult health by utilising unpublished cohort data on cardiovascular disease in individuals with and without a history of out-of-home care in childhood that was captured prospectively. In doing so, we assess if the relationship is confounded by family social circumstances, mediated by adult health behaviour (cigarette smoking) or social status, and whether the care–cardiovascular disease association varies according to key contexts, including sex, age at care entry, and country.

## Methods

This is a systematic review with meta-analysis. The protocol was prospectively registered (PROSPERO CRD42021254665) and this manuscript was constructed in accordance with the Meta-analysis Of Observational Studies in Epidemiology (MOOSE) guidelines.^26^ We took two approaches to identifying relevant data on childhood care and adult cardiovascular disease. First, to locate published studies, we searched PubMed (Medline). Second, to identify unpublished datasets with the potential to address the present research question, we scrutinised the reference section of retrieved papers.

### Identifying published data

The PubMed database was searched by GDB from its inception in 1966 to November 21, 2023. Without applying any restrictions, we used a series of terms for the exposure (e.g., ‘out-of-home care’, ‘foster care’, ‘public care’, ‘looked after children’) and the outcome (e.g., ‘cardiovascular disease’, ‘coronary heart disease’, ‘stroke’) in the context of longitudinal studies (e.g., ‘cohort’, ‘follow-up’). For all search terms see Supplemental File S1. For inclusion, studies needed to satisfy three criteria: a cohort study in which the assessment of care was made prospectively pre-adulthood (in the avoidance of recall bias); data on an unexposed comparator group were available (for the computation of relative risk); and a diagnosis of adult cardiovascular disease events (coronary heart disease, stroke, or their combination) had been made (as opposed to risk factors or risk score). Duplicate studies were collapsed into a single record. The outcome of study screening was corroborated by PF; there were no disagreements. Details of included studies—study name, authors, sample size, number of cardiovascular disease events—were subsequently into a database. Requests were then made to authors for analyses based on updated cardiovascular disease event surveillance as appropriate.

### Identifying unpublished data

GDB scrutinised the reference section of retrieved papers to identify studies with the potential to examine the relationship between childhood care and adult cardiovascular disease. In practice, this was those studies relating childhood care to adult health and health-orientated outcomes such as mental illness or socio-economic circumstances. Unpublished studies had to satisfy the same inclusion criteria as those for published studies.

At this stage, only the available basic study characteristics—author name, contact details, study name—were then entered into the same database as those for the published studies. Once potential collaborators had been contacted and confirmed the availability of the required data, an analytical plan was circulated (Supplemental File S2). The same information was required as that for published studies. Analysis of the relation between care and adult cardiovascular disease was conducted using either time-to-event analyses or logistic regression as per the available data. New results were entered into the database as study teams returned them. Data provision resulted in authorship of up to two study investigators, typically the principal investigator and the data analyst.

In the guidance for authors, three sources of cardiovascular disease data were identified as being acceptable. First, registry data for death or hospitalisations from which International Classification of Disease (ICD) codes for coronary heart disease (ICD-9: 410–414; ICD-10: I20–25) and stroke (ICD-9: 430–438; ICD-10: I60–69) could be extracted. Mortality and hospital records (morbidity) for cardiovascular disease can be used interchangeably.^27^ Second, medical examination for coronary heart disease (e.g., electrocardiogram, raised cardiac enzyme activity) and stroke (e.g., computerised tomography scan, magnetic resonance imaging). Third, self-report of a relevant medical condition (e.g., heart attack, myocardial infarction, angina; cerebrovascular disease/accident) or a medical procedure (e.g., coronary artery bypass graft, percutaneous coronary intervention). Self-reported coronary heart disease (kappa statistic 0.70) and stroke (0.66) show a level of agreement with hospital records that is acceptable for the purposes of population-based research.^28^

Where the data were available, we requested that investigators statistically adjust effect estimates for potentially important explanatory factors in their analyses. Confounding factors included early life socioeconomic status as indexed by parental occupational social class, education, or income, with the substitution of area-based measures if these individual-level data were unavailable. Potential mediating variables requested included health behaviours (e.g., cigarette smoking) and socioeconomic status—both captured in adulthood.

### Evaluation of study quality

GDB and PF used the Newcastle-Ottawa Scale to appraise the quality of each study (Supplemental File S1).^29^ For published studies, existing reports were assessed; for unpublished studies, we made use of a combination of publications in which the ascertainment of childhood care or cardiovascular disease was described plus any other supporting documentation provided by the authors. Comprising eight domains, including the comprehensiveness of exposure and outcome ascertainment and adequacy of the period of health surveillance, a higher score on this scale denotes higher study quality (maximum score 9). For the purposes of the present review, studies with a score of 7 or more on the Newcastle-Ottawa Scale were denoted as being of high grade.

### Statistical analyses

For individual studies using time-to-event analyses, hazard ratios with accompanying 95% confidence intervals were computed using Cox regression.^30^ Where these data were not available, logistic regression was used to calculate odds ratios. When the cumulative event incidence is 10% or less of the study sample, as is the case in the present analyses for cardiovascular disease in populations censored by middle-age, the odds ratios and hazards ratios closely approximate for the range of study-specific effect estimates reported.^31^

Initially, in the model with basic adjustment, we explore the impact of confounding by controlling for age, sex, or their combination; data from birth cohort studies where members were born within a single week did not require age adjustment. Family socio-economic status in early life was then added to this model. Next, we examined the role of mediation by social circumstances and cigarette smoking in adulthood. In all these analyses, we observed the change in the risk ratio from basic adjustment after each explanatory variable—confounder or mediator—was added to the multivariable model in a non-accumulative manner. These study-specific results were pooled using a random effects meta-analysis based on the DerSimonian-Laird method,^32^ an approach which incorporates the heterogeneity of study-level results into the computation of their aggregation. An *I*^*2*^ statistic was computed to summarise this heterogeneity. To examine the robustness of our findings, we explored the magnitude of the care–cardiovascular disease association (basic adjustment) according to different contexts, including sex, study quality, and geographical region. All analyses were computed using Stata version 17 (StataCorp, College Station, TX), R version 4.3.1, or RStudio version 2023.03.1.

### Role of the funding source

The funding source had no role in design, analysis, interpretation, or report writing for this study.

## Results

**Fig. 1:**
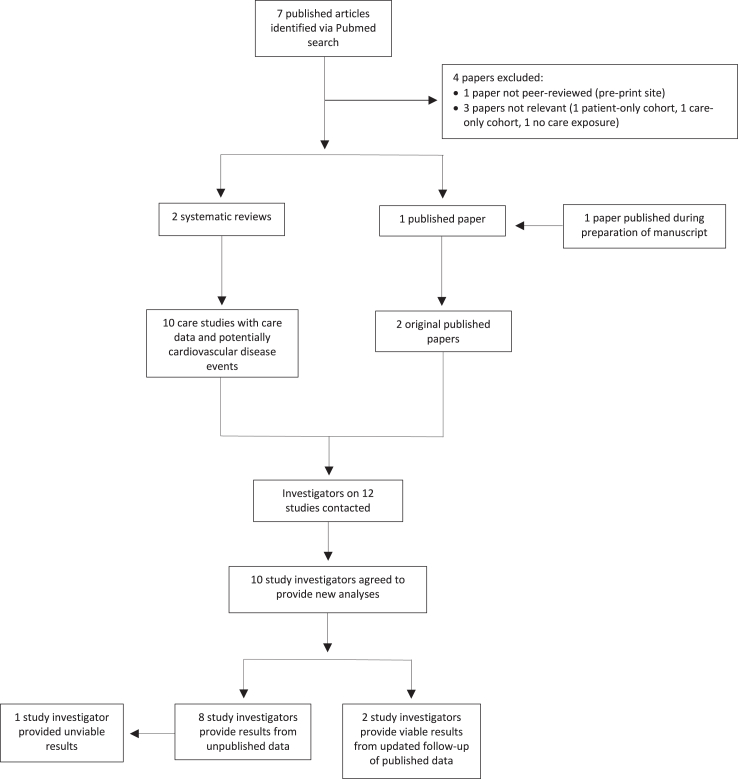
Identification of published and unpublished studies on childhood care and adult cardiovascular disease.

A search of electronic databases revealed 1 published study^19^ matching our inclusion criteria, while another was published by collaborators during the preparation of this manuscript^20^ (Fig. 1). Additionally, using citations in published systematic reviews,^18^^,^^33^ we identified ten unpublished studies that had the potential to examine the relation between childhood care and adult cardiovascular disease. In combination, this resulted in 12 seemingly unique datasets.^16^^,^^19^^,^^20^^,^^34^, ^35^, ^36^, ^37^, ^38^, ^39^, ^40^, ^41^, ^42^ Requests for collaboration yielded 10 positive responses and 9 study investigators provided viable results which included updated analyses of the two published studies.

Individual study characteristics are given in Table 1. Seven studies were based on samples drawn from Europe^16^^,^^19^^,^^20^^,^^35^^,^^37^^,^^41^^,^^42^ with an additional two from the USA^39^ and Australia.^34^ There was some variation in data collection methods used across studies. Four studies relied on parent/carer reported care history,^16^^,^^35^^,^^39^^,^^41^ while 5 utilised registry data on this exposure.^19^^,^^20^^,^^34^^,^^37^^,^^42^ In three cohorts, study members self-reported a physician diagnosis of cardiovascular disease,^16^^,^^35^^,^^39^ and in the remaining 6 physician-verified cardiovascular disease hospitalisations and/or deaths were extracted from national or regional registries.^19^^,^^20^^,^^34^^,^^37^^,^^41^^,^^42^ With the exception of the iCAN study which used an area-based indicator,^34^ socioeconomic status was captured at the level of individual in early and later life using indices which included occupational social class,^19^ educational attainment,^42^ and receipt of welfare payments.^39^ Taken together, five of the nine studies were judged as being of higher methodological quality (Supplemental File S1).^19^^,^^20^^,^^37^^,^^41^^,^^42^

**Table 1:** Characteristics of studies included in the meta-analysis.

Study^key citation^ (country)	Year of birth	Number of participants (women)	Age at care assessment (years)	Care ascertainment	Proportion in care, N (%)	SES indicator in childhood (adulthood)	Maximum age at follow-up (years)	Total number of CVD events	CVD ascertainment
Helsinki Birth Cohort Study^19^ (Finland)	1934–1944	12,951 (6119)	0–11	Register	13.3	Parental social class (own social class)	84	3578	Registers of hospitalisations and deaths
Stockholm Birth Cohort Study^20^ (Sweden)	1953	14,543 (7135)	0–18	Register	9.1	Parental social class (own education)	65	1081	Registers of hospitalisations and deaths
Office for National Statistics Longitudinal Study^41^ (UK)	1953–2001	353,601 (172,696)	1–17	Carer report	1.7	Parental social class (own social class)	60	503	Register of deaths
1958 British Birth Cohort Study^16^ (UK)	1958	8488 (4371)	7–16	Parental report	3.2	Parental social class (own social class)	58	457	Self-report
Woodlawn Cohort Study^39^ (USA)	1960	1053 (549)	6–7	Parental report	1.4	Mother’s welfare payment (own welfare payment)	44	34	Self-report
1970 British Birth Cohort Study^35^ (UK)	1970	7888 (4072)	5–16	Parental report	4.7	Parental social class (own education)	43	203	Self-report
impacts of Child Abuse and Neglect (iCAN) South Australia Cohort Study^34^ (Australia)	1972–1999	94,799 (43,851)	0–18	Register	6.6	Not available (own neighbourhood)	45	255	Registers of hospitalisations, deaths & emergency room visits
1987 Finnish Birth Cohort Study^42^ (Finland)	1987	59,476 (29,041)	0–18	Register	3.2	Parental education (own education)	33	358	Registers of hospitalisations and deaths
1997 Finnish Birth Cohort Study^37^ (Finland)	1997	58,802 (28,924)	0–18	Register	5.7	Parental education (own education)	23	66	Registers of hospitalisations and deaths

SES, socioeconomic status; NR, not report; UK, United Kingdom; and CVD, cardiovascular disease. Studies are ordered by ascending birth year. The iCAN study comprises a Child Protection cohort (record in child protection system from 1986) and a birth cohort (those born from 1986); prior publications have been based on the birth cohort only.

In total, the nine included studies comprised 611,601 individuals (301,129 women), with individual cohort size ranging from 1053^39^ to 353,601.^41^ Births occurred across eight decades (1934^19^ to 2001^41^). The period prevalence of out-of-home care in childhood varied from 1.4% (USA)^39^ to 13.3% (Finland).^19^ There was a total of 6535 cases of cardiovascular disease in adulthood, the most in a single study being 3578 such events.^19^ The maximum age at follow-up was 84 years.^19^

**Fig. 2:**
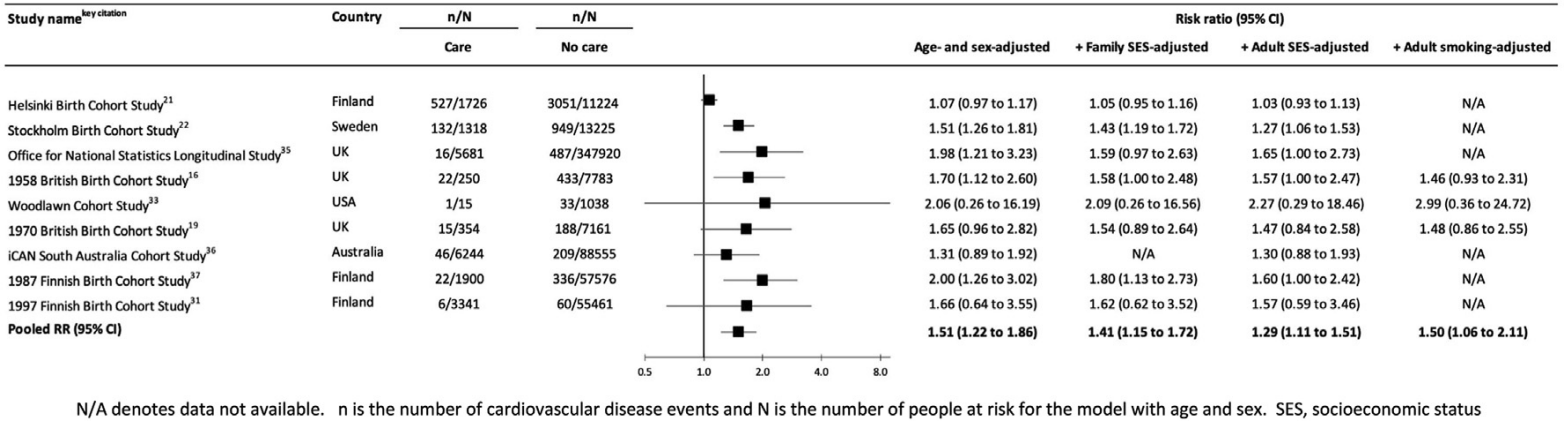
Association between public care in childhood and risk of cardiovascular disease in adulthood.

In Fig. 2 we show the study-specific associations between care ascertained in childhood and subsequent adulthood cardiovascular disease risk. After the basic adjustments—age alone, sex alone, or a combination—in each of the 9 included studies, the point estimates indicated that a history of care placement during childhood was related to an elevated risk of adulthood cardiovascular disease. While these study-specific risk ratios were directionally consistent and above unity, there was heterogeneity in their magnitude (range: 1.07 to 2.06, I^2^ 69%, p-value 0.001), and in five studies the care–cardiovascular disease association was not statistically significant at conventional levels. Aggregating these estimates resulted in a 51% increase in the risk of cardiovascular disease in adults with a history of out-of-home care in childhood (1.51 [1.22, 1.86]).

Next, we examined the role of confounding and mediating factors in the care–cardiovascular disease association. There was marginal attenuation of the care–cardiovascular disease relation after taking into account childhood (family) social circumstances (8 studies, 1.41; 1.15, 1.72). When study members’ own socioeconomic status in adulthood, a potential mediator, was added to the multivariable model, however, the relationship between care and cardiovascular disease was markedly attenuated (9 studies, 1.29; 1.11, 1.51). In contrast, controlling for adult cigarette smoking was not indicative of mediation (1.50; 1.06, 2.11) although this was based on only 3 studies with these data.

**Fig. 3:**
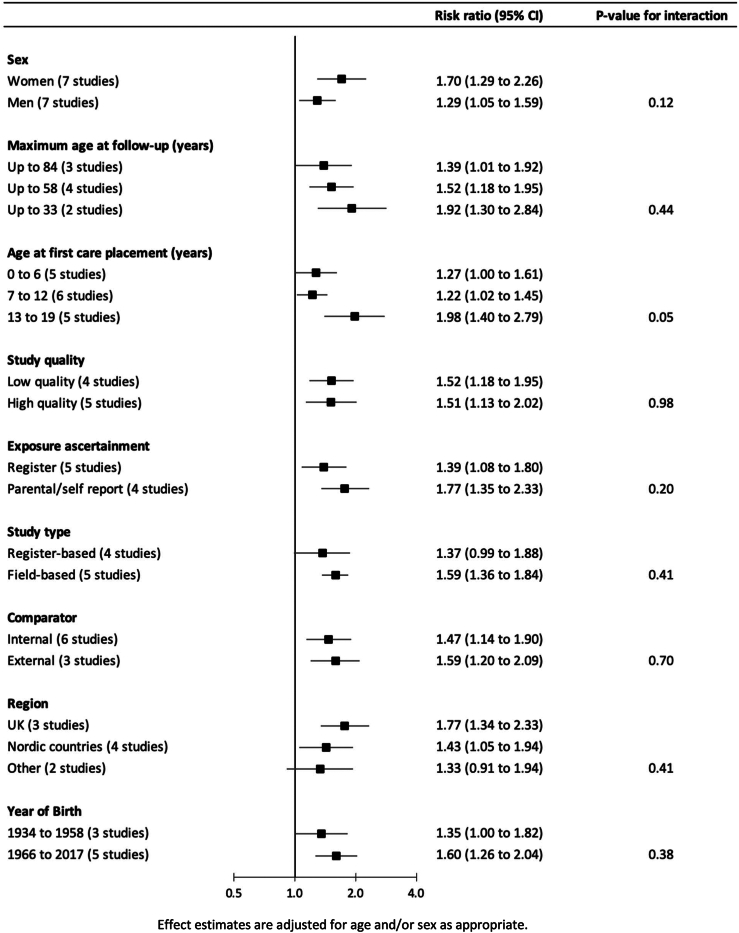
Association between public care in childhood and risk of cardiovascular disease in adulthood according to context.

Results for the impact, if any, of different contexts on the care–cardiovascular disease relation are shown in Fig. 3. With the exception of age at care placement (p-value for interaction = 0.05), while there were some differences in the magnitude of the care–cardiovascular disease association, these were not statistically significant at conventional levels. Thus, marginally stronger associations were apparent in study participants who were placed in out-of-home care later in childhood (1.98; 1.40, 2.79) relative to earlier (1.27; 1.00, 1.61), and in women (1.70; 1.29, 2.26) than men (1.29; 1.05, 1.59).

We carried out some *post hoc* sensitivity analyses. In the Helsinki Birth Cohort,^19^ children were evacuated from Finland to the ostensibly safer country of Denmark during World War II. This contrasts with the circumstances of removal from the biological family in other studies featured in our review, such that Finnish parents volunteered their children for evacuation owing to an abundance of concern for their safety as opposed to them being removed by the state. After excluding this study result from our main analyses, the pooled result based on 8 studies was little affected, however (1.59; 1.39, 1.81). Second, the sole US cohort—the Woodlawn Cohort Study^39^—was also somewhat of an outlier based on both the prevalence of care (1.4%), the lowest of the recruited studies, while the risk ratio for care and cardiovascular disease was the highest (2.06; 0.26, 16.19). This sample was drawn from an African American community residing in a socioeconomically disadvantaged neighbourhood in a large urban conurbation (Chicago). On excluding this study from the main analyses, this new pooled age- and sex-adjusted risk ratio (1.50 [1.21, 1.86]) was, again, immaterially different from that based on all studies. Lastly, we repeated the main meta-analysis using Mantel-Haenszel instead of inverse variance weighting and the rate ratio was of lower magnitude (1.27 [1.19, 1.35]).

## Discussion

The main finding of this meta-analysis of unpublished results from nine cohort studies was that adults with a history of care placement in childhood had a moderately raised risk of later cardiovascular disease. The magnitude of this association is commensurate with childhood overweight, cigarette smoking, and raised levels of blood pressure and blood cholesterol.^7^, ^8^, ^9^, ^10^, ^11^, ^12^, ^13^, ^14^ While adjusting for childhood socioeconomic status and adult cigarette smoking had little impact on this association, there was marked attention by social circumstances in later life. This may indicate that state care places an individual on a trajectory of socioeconomic disadvantage which extends into adult life, an observation made elsewhere when total mortality was the endpoint of interest.^43^

Although there was no strong evidence of interaction according to any of the contexts we examined, our finding of a marginally stronger relationship of placement in care with cardiovascular disease in women than men was also apparent in a recent systematic review in which completed suicide was the outcome of interest.^18^ These results perhaps run counter to speculation that girls are more resilient to stressful early life circumstances than boys.^44^ The observation of sensitive periods of exposure—we found marginally stronger associations with cardiovascular disease in people who entered care later in childhood—has been made in relation to other health outcomes, including all-cause mortality, and has an array of plausible explanations.^18^ Older age at care entry could, for instance, simply be a proxy for extended exposure to a dysfunctional home environment. Relatedly, the reasons for care initiation seem to vary by age, such that parental abuse is more common in children entering at younger ages, while behavioural issues (e.g., delinquency) become more prevalent in adolescence.^45^ Differences in care according to country were examined because the composition of this exposure will vary: whereas in the UK the system is largely based on permanent removal of children from families for the child’s protection, an action that is often against the wishes of biological parents, in the Nordic countries out-of-home care is more likely to be a temporary family support measure.^46^ While there was a suggestion of a stronger care–cardiovascular disease association in the UK than in the Nordic countries and South Australia, which has similar care policies, again, these differentials did not attain statistical significance at conventional levels.

A strength of our meta-analysis is its relative novelty—at the time of the database search, only one cohort had examined the association between prospectively measured exposure to childhood care and adult cardiovascular disease. As described, agreement between retrospective and prospective (gold standard) assessments of childhood maltreatment is poor.^23^ There are obvious reasons to expect several biases to exert an impact on the quality of recalled data elicited many years following adverse events, including simply the protective mechanism of forgetting and the role of life events in the intervening years such that an individual with contemporary experience of chronic illness particularly mental ill-health, may not provide the same unbiased account of early life misery as a person who is illness-free.

Our meta-analysis is of course not without its shortcomings. First, while there was some evidence of mediation by adult social circumstances, of the health behaviours, we only had data on adult smoking but not physical activity nor alcohol intake. The lack of data on candidate biological mediators, including markers of metabolic, immune, neuroendocrine, and autonomic functioning may not be a limitation, however, given that these characteristics were not related to earlier care exposure in two of the birth cohorts featured here.^35^^,^^47^ For some of the collected data, there was also some heterogeneity. Socioeconomic status, for instance, was captured using a range of indices (i.e., occupational social class, education, welfare receipt, area-based indicators). However, despite acting at different points in the life course, inter-correlations coefficients for these measures are moderately-high and directionally consistent and, as result, are associated with mortality in a similar fashion.^48^^,^^49^

Second, with the included studies being observational, the standard caveat about cause and effect applies. An alternative approach to addressing the present question that would circumvent the primary concern of confounding is a randomised controlled trial in which half of children requiring transfer to a safer environment would be allocated to state care while the rest remain with their family of origin. With such a trial being potentially unethical, a further option is a natural experiment whereby the impact of changes in state care policy (e.g., to reduce the number of children being placed in out of the home) on cardiovascular disease risk is explored. Analyses of siblings or twins who are discordant for the exposure, whereby one child is taken into care but the other remains with the biological family, would also have utility.

Third, while we were able to examine the association of age at care entry with cardiovascular disease, we did not have data on other potentially important care characteristics such as reason for removal to care (e.g., disability, child health), care type (e.g., foster care, institution), and duration across a sufficiently large number of studies to facilitate analyses. That we found marked cross-study differences in care–cardiovascular disease effect estimates could be ascribed to heterogeneity in exposure such that there is variation in care type (e.g., foster home versus institution-based). Fourth, quantification of the long-term health consequences of pre-adult care necessarily requires cohort studies in which the type of care being examined may no longer be that which occurs in the present day. This raises the issue of extrapolation of these historical results to current public policy. Fifth, we attempted to disentangle the impact of pre-care trauma from the effect of care itself by controlling for childhood socioeconomic circumstances. While this had little impact on the magnitude of the care–cardiovascular disease relationship, the utility of these data for this purpose is low. Lastly, with some exceptions,^34^^,^^39^ included study samples comprised largely ethnically white study participants. While it is unlikely that the care–cardiovascular disease gradient in minority groups would be directionally inconsistent with the present results, empirical testing is perhaps warranted.

### Conclusions

Our findings from a pooling of nine unpublished cohorts from the US, UK, Sweden, Finland, and Australia show that individuals who experienced out-of-home care during childhood had a moderately elevated risk of cardiovascular disease in adulthood. For children with a care history who are therefore known to health and social services, it may be that existing protections are insufficient to address the burden of this chronic disease.

## Contributors

GDB generated the idea for this project, conducted the literature search, contacted study principal investigators, developed an analytical plan, collated results, evaluated study quality, and drafted the manuscript. PF corroborated the outcomes of the literature search and study quality, conducted the meta-analysis, prepared the figures, and edited the manuscript. MK contacted study principal investigators, developed an analytical plan, and edited the manuscript. ESG, MH, JJ, H-SJ, MK-S, CL, TMM and AS analysed individual participant data and edited the manuscript. YBA, JGE, MG, EM, and LS edited the manuscript. GDB and PF accessed and verified the study-specific results. All authors were responsible for the decision to submit the manuscript.

## Data sharing statement

Bona fide researchers interested in individual study datasets included in this meta-analysis should contact individual study investigators, not GDB.

## Declaration of interests

None.
