# Supplementary material for: Cardiovascular disease in adults with a history of out-of-home care during childhood: a systematic review and meta-analysis of prospective cohort studies
Source: Lancet Reg Health Eur. 2024 Jul 9;43:100984. doi: 10.1016/j.lanepe.2024.100984 (PMC11284711; doi:10.1016/j.lanepe.2024.100984)
Supplement: Supplemental File S1 [file mmc1.docx]

**Supplemental File 1 - Search string for PubMed and study quality assessment**

**Batty et al. Frank. Cardiovascular Disease Events in Adults with a History of State Care in Childhood: Pooling of Unpublished Results from 9 Cohort Studies**

**Supplemental Box 1. Search string for PubMed (Medline)**

| #1 (cardiovascular disease[Title/Abstract]) OR (coronary artery disease[Title/Abstract]) OR (coronary heart disease[Title/Abstract]) OR (myocardial infarction[Title/Abstract]) OR (ischemic heart disease[Title/Abstract]) OR (ischaemic heart disease[Title/Abstract]) OR (acute coronary syndrome[Title/Abstract]) OR (stroke[Title/Abstract]) OR (cerebrovascular accident[Title/Abstract]) OR (cerebrovascular disease[Title/Abstract]) OR (cardiovascular events[Title/Abstract]) OR (cardiovascular deaths[Title/Abstract]) OR (heart failure[Title/Abstract])  #2 (out-of-home care [Title/Abstract]) OR (out of home care [Title/Abstract]) OR (foster care [Title/Abstract]) OR (public care [Title/Abstract]) OR (looked-after-children [Title/Abstract]) OR (looked after children[Title/Abstract])  #3 (epidemiologic studies[MeSH Terms]) OR (cohort studies[MeSH Terms]) OR (epidemiologic[Text Word]) OR (longitudinal[Text Word]) OR (cohort[Text Word]) OR (follow up[Text Word]) OR (observational[Text Word]) OR (prospective[Text Word])  #4 #1 AND #2 AND #3 |
| --- |

**Supplemental Table 1. Cohort study quality assessment according to the**

**Newcastle-Ottawa criteria: Meta-analysis**

| **Study name^key citation^** | **Selection** | | | | **Comparability** | **Outcome** | | | **Total quality score (0-9)** |
| --- | --- | --- | --- | --- | --- | --- | --- | --- | --- |
|  | **Representative(0-1)** | **Selection – unexposed**  **(0-1)** | **Ascertainment exposure (0-1)** | **Outcome absent at baseline (0-1)** | **Case/control comparability**  **(0-2)** | **Assessment of outcome (0-1)** | **Length of follow-up**  **(0-1)** | **Adequacy of follow-up**  **(0-1)** |  |
|  |  |  |  |  |  |  |  |  |  |
| Helsinki Birth Cohort Study^1^ | 0 | 1 | 1 | 1 | 2 | 1 | 1 | 1 | 8 |
| Stockholm Birth Cohort Study^2^ | 0 | 1 | 1 | 1 | 2 | 1 | 1 | 1 | 8 |
| Office for National Statistics Longitudinal Study^3^ | 1 | 1 | 1 | 1 | 2 | 1 | 1 | 0 | 8 |
| 1958 British Birth Cohort Study^4^ | 1 | 1 | 0 | 1 | 1 | 0 | 1 | 1 | 5 |
| Woodlawn Cohort Study^5^ | 1 | 1 | 0 | 1 | 1 | 0 | 1 | 0 | 5 |
| 1970 British Birth Cohort Study^6^ | 1 | 1 | 0 | 1 | 1 | 0 | 1 | 0 | 5 |
| iCAN South Australia Cohort Study^7^ | 0 | 1 | 1 | 1 | 2 | 1 | 0 | 0 | 6 |
| 1987 Finnish Birth Cohort Study^8^ | 1 | 1 | 1 | 1 | 2 | 1 | 0 | 0 | 7 |
| 1997 Finnish Birth Cohort Study^9^ | 1 | 1 | 1 | 1 | 2 | 1 | 0 | 0 | 7 |
|  |  |  |  |  |  |  |  |  |  |

A higher score denotes higher study quality

**References**

1. Alastalo H, Raikkonen K, Pesonen AK, et al. Cardiovascular morbidity and mortality in Finnish men and women separated temporarily from their parents in childhood--a life course study. *Psychosom Med* 2012; **74**(6): 583-7.

2. Jackisch J, Almquist YB. Childhood adversity is associated with hospitalisations and survival following external causes and non-communicable diseases: a 46-year follow-up of a Stockholm birth cohort. *J Epidemiol Community Health* 2023; **77**(4): 209-15.

3. Murray ET, Lacey R, Maughan B, Sacker A. Association of childhood out-of-home care status with all-cause mortality up to 42-years later: Office of National Statistics Longitudinal Study. *BMC Public Health* 2020; **20**(1): 735.

4. Xie TH, de Mestral C, Batty GD. Association of public care in childhood with social, criminal, cognitive, and health outcomes in middle-age: six decades of follow-up of members of the 1958 Birth Cohort Study. *medRxiv* 2020.

5. Juon HS, Evans-Polce RJ, Ensminger M. Early life conditions of overall and cause-specific mortality among inner-city African Americans. *Am J Public Health* 2014; **104**(3): 548-54.

6. Batty GD, Hamer M. Public care during childhood and biomedical risk factors in middle-age: the 1970 birth cohort study. *Am J Epidemiol* In press.

7. Segal L, Nguyen H, Mansor MM, et al. Lifetime risk of child protection system involvement in South Australia for Aboriginal and non-Aboriginal children, 1986-2017 using linked administrative data. *Child Abuse Negl* 2019; **97**: 104145.

8. Sariaslan A, Kaariala A, Pitkanen J, et al. Long-term Health and Social Outcomes in Children and Adolescents Placed in Out-of-Home Care. *JAMA Pediatr* 2022; **176**(1): e214324.

9. Kalland M, Pensola TH, Merilainen J, Sinkkonen J. Mortality in children registered in the Finnish child welfare registry: population based study. *Bmj* 2001; **323**(7306): 207-8.
